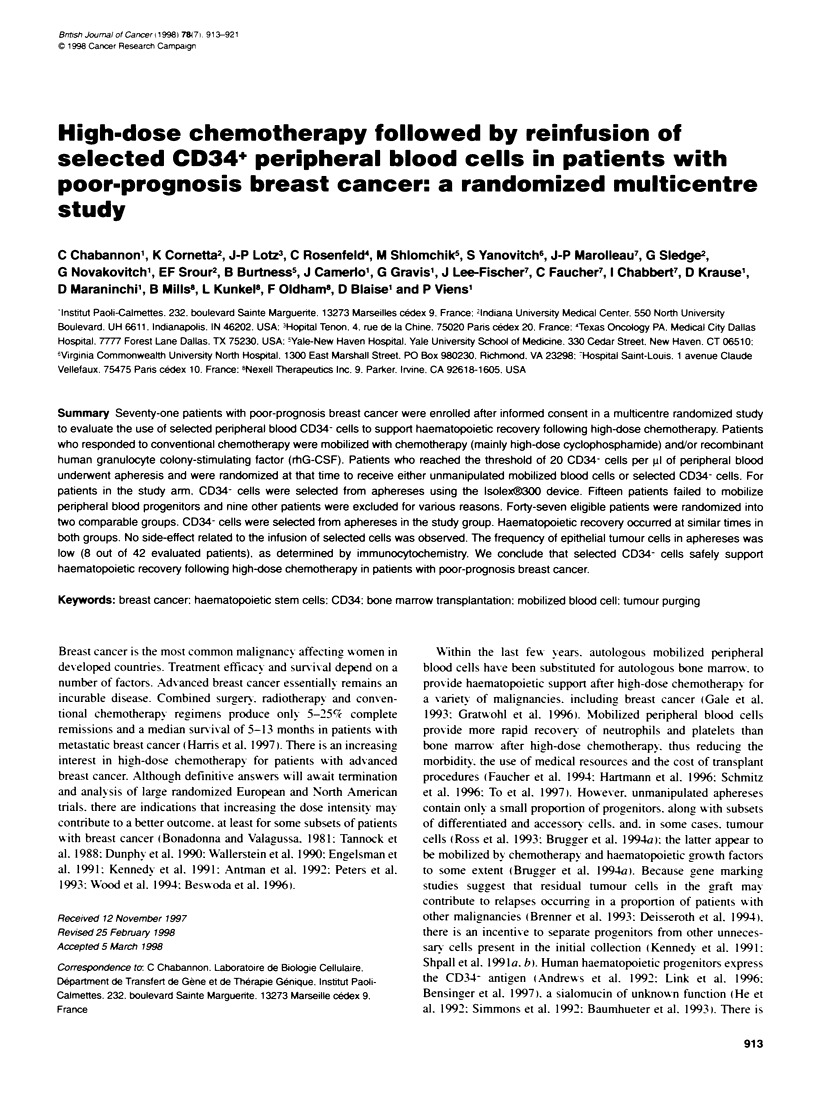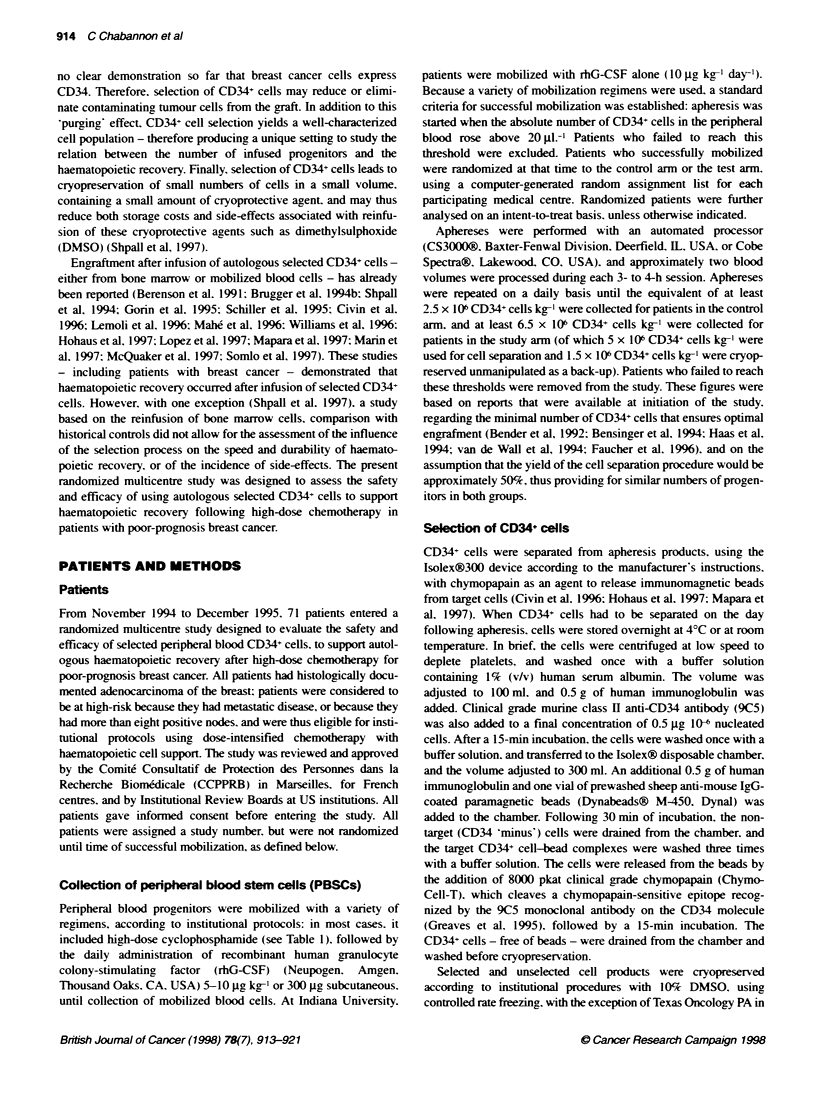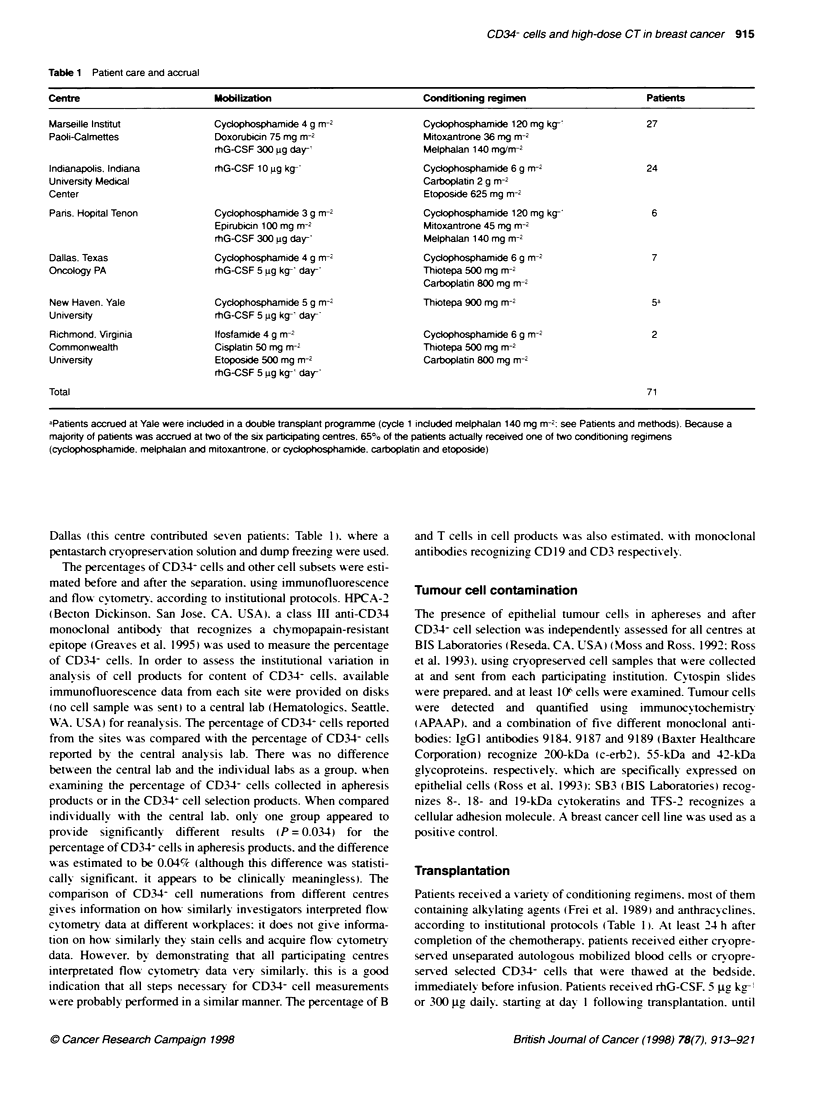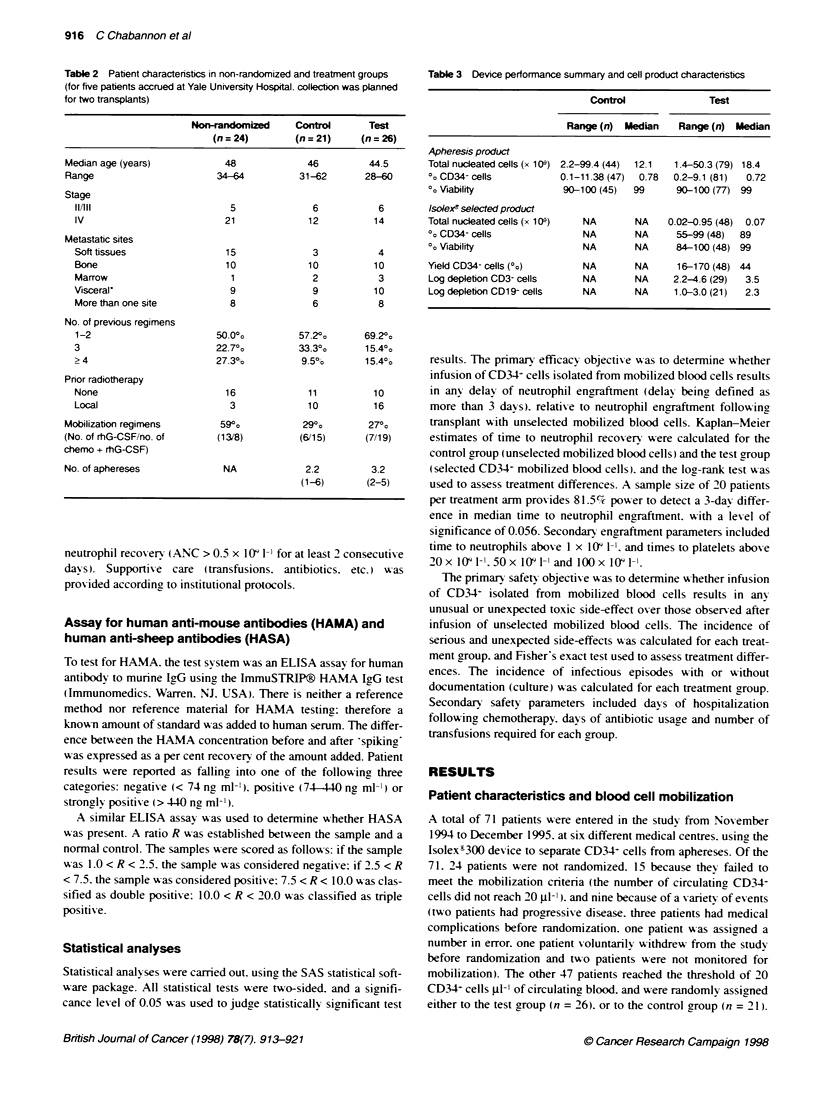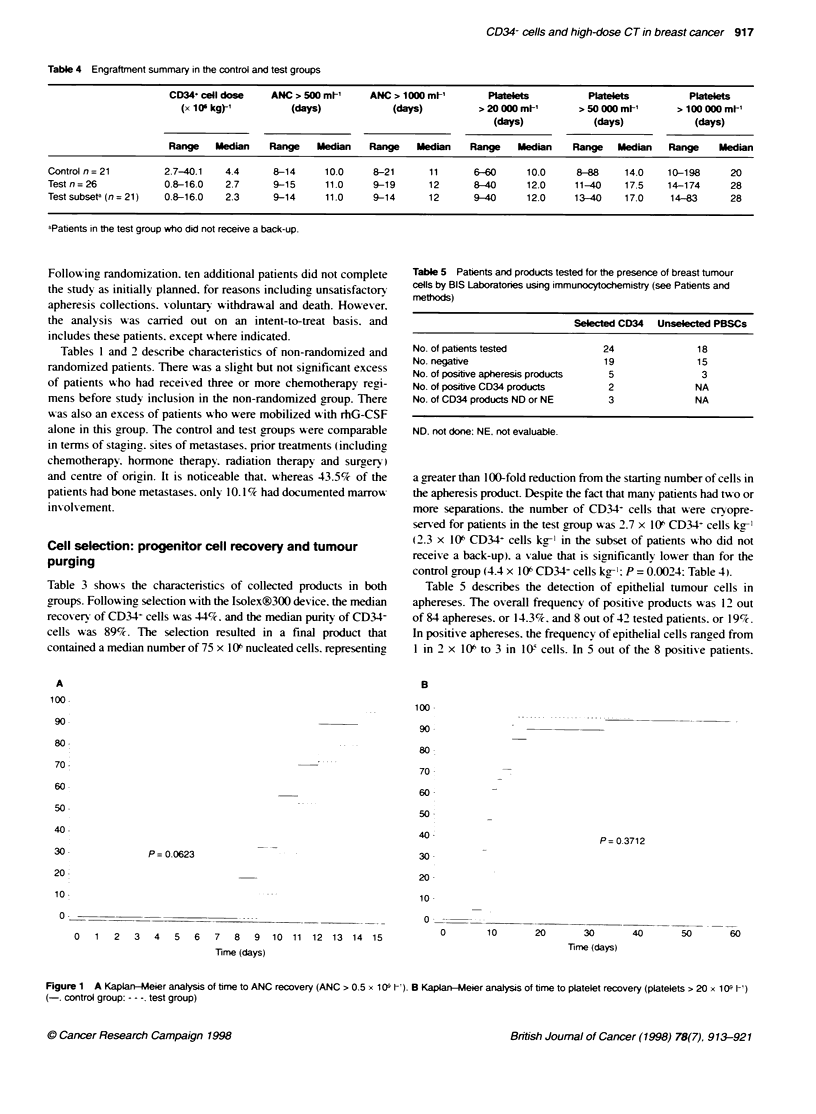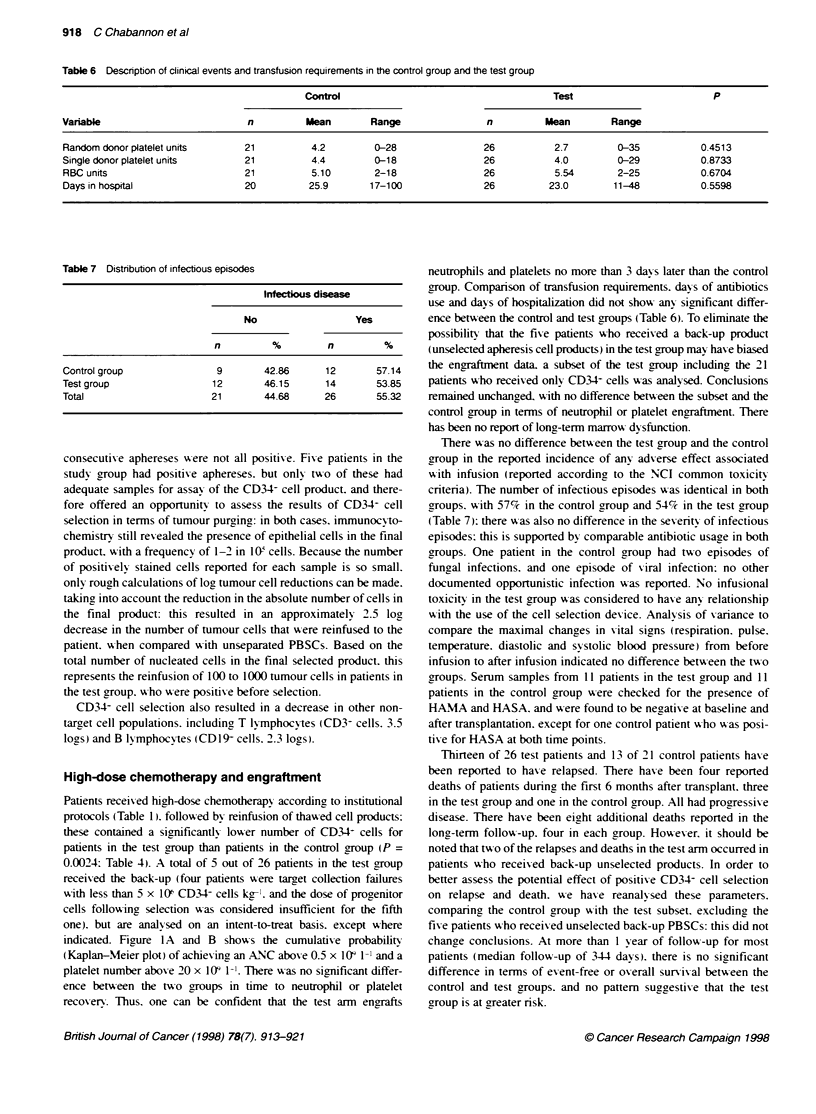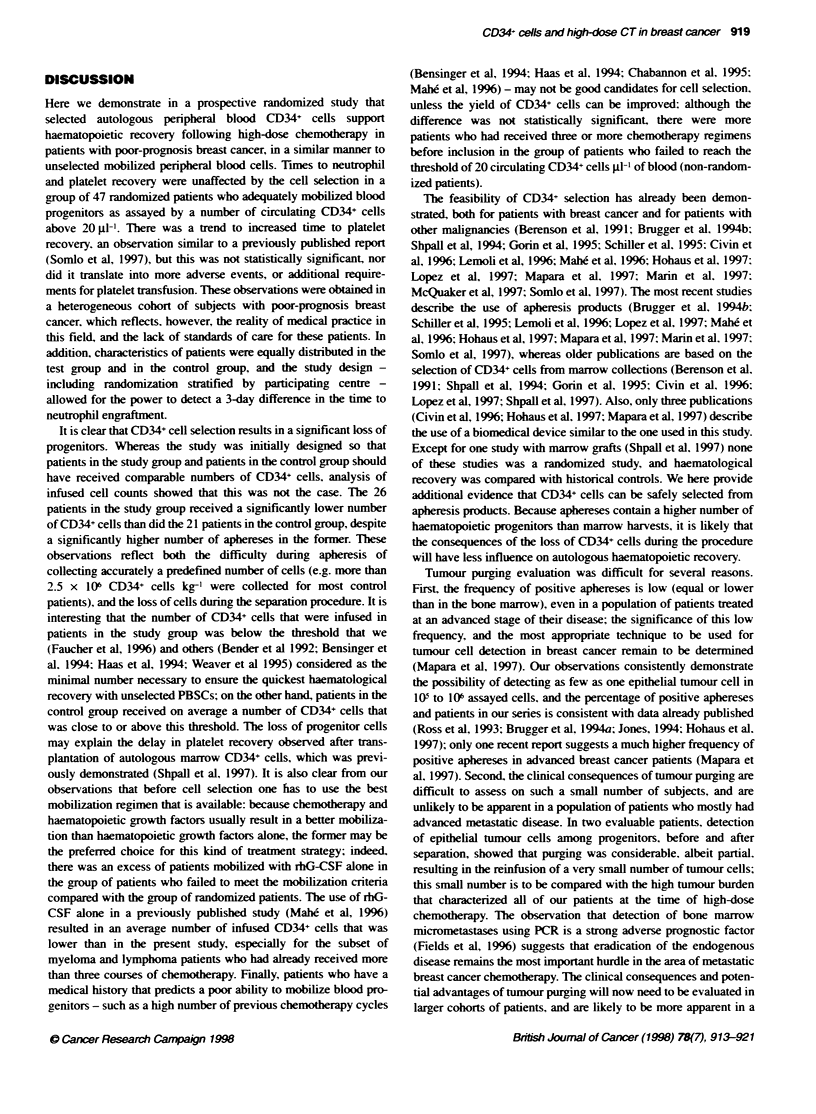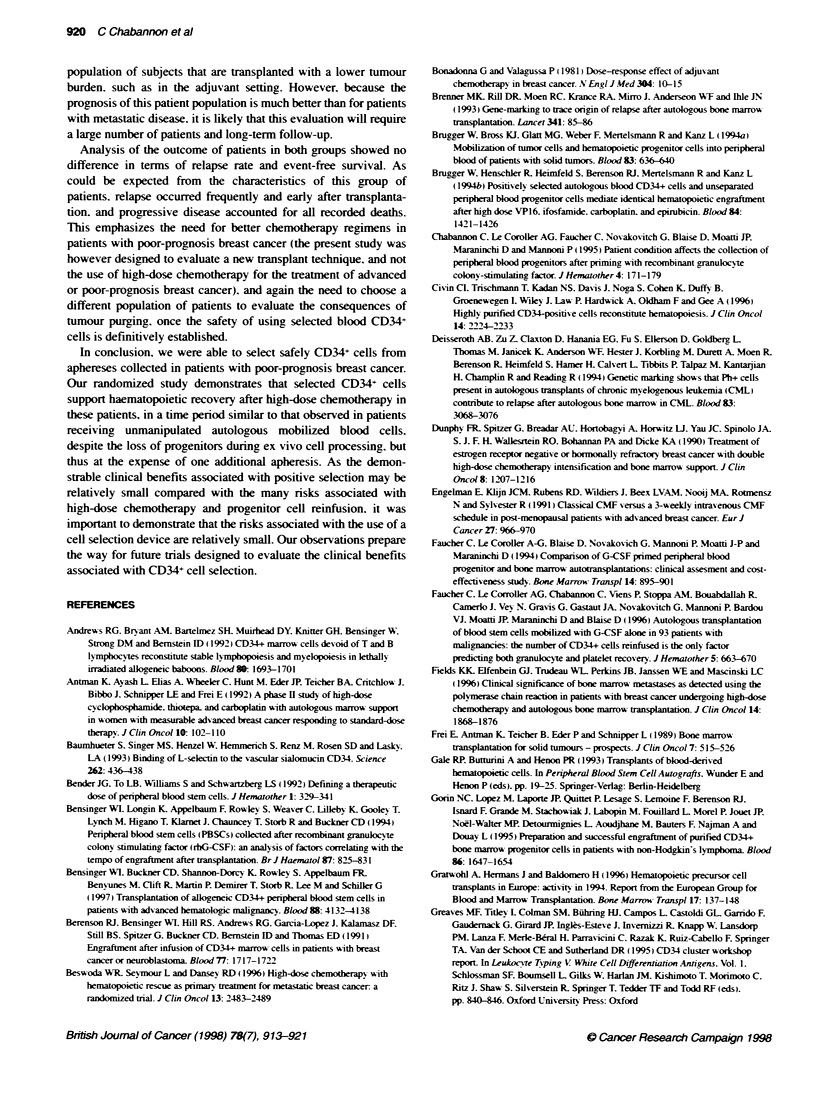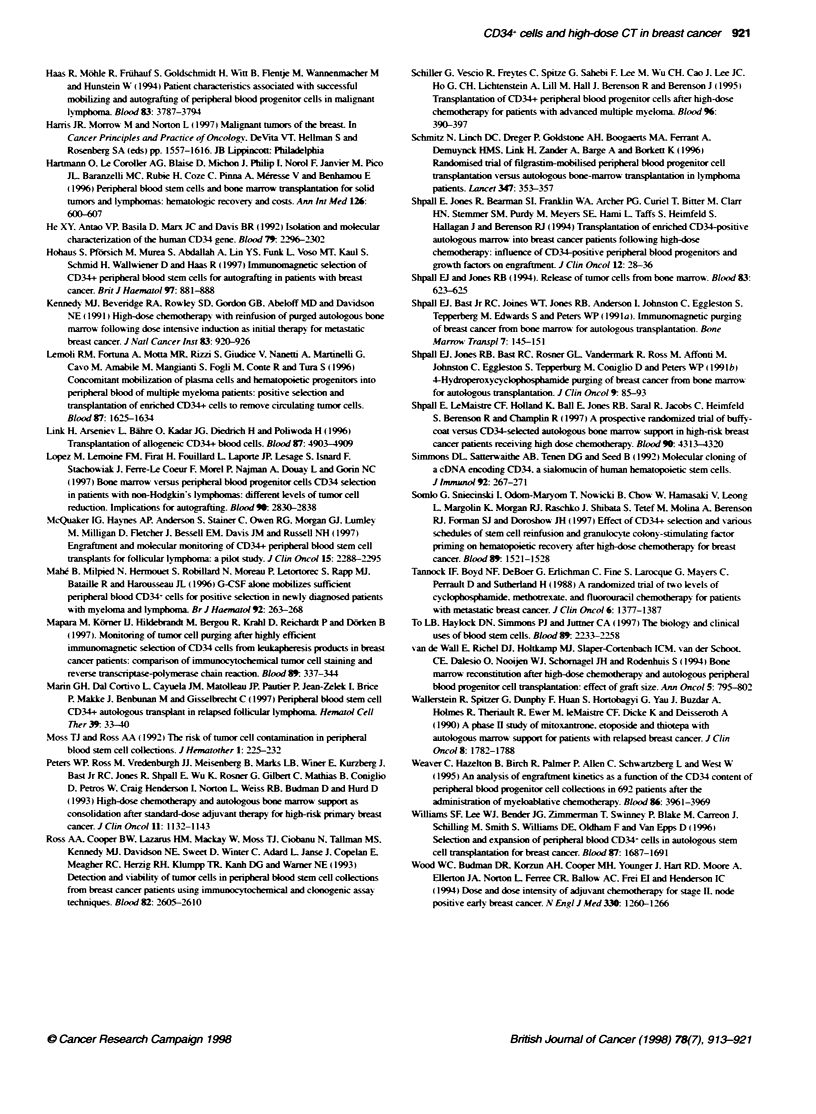# High-dose chemotherapy followed by reinfusion of selected CD34+ peripheral blood cells in patients with poor-prognosis breast cancer: a randomized multicentre study.

**DOI:** 10.1038/bjc.1998.601

**Published:** 1998-10

**Authors:** C. Chabannon, K. Cornetta, J. P. Lotz, C. Rosenfeld, M. Shlomchik, S. Yanovitch, J. P. Marolleau, G. Sledge, G. Novakovitch, E. F. Srour, B. Burtness, J. Camerlo, G. Gravis, J. Lee-Fischer, C. Faucher, I. Chabbert, D. Krause, D. Maraninchi, B. Mills, L. Kunkel, F. Oldham, D. Blaise, P. Viens

**Affiliations:** Institut Paoli-Calmettes, Marseilles, France.

## Abstract

Seventy-one patients with poor-prognosis breast cancer were enrolled after informed consent in a multicentre randomized study to evaluate the use of selected peripheral blood CD34+ cells to support haematopoietic recovery following high-dose chemotherapy. Patients who responded to conventional chemotherapy were mobilized with chemotherapy (mainly high-dose cyclophosphamide) and/or recombinant human granulocyte colony-stimulating factor (rhG-CSF). Patients who reached the threshold of 20 CD34+ cells per microl of peripheral blood underwent apheresis and were randomized at that time to receive either unmanipulated mobilized blood cells or selected CD34+ cells. For patients in the study arm, CD34+ cells were selected from aphereses using the Isolex300 device. Fifteen patients failed to mobilize peripheral blood progenitors and nine other patients were excluded for various reasons. Forty-seven eligible patients were randomized into two comparable groups. CD34+ cells were selected from aphereses in the study group. Haematopoietic recovery occurred at similar times in both groups. No side-effect related to the infusion of selected cells was observed. The frequency of epithelial tumour cells in aphereses was low (8 out of 42 evaluated patients), as determined by immunocytochemistry. We conclude that selected CD34+ cells safely support haematopoietic recovery following high-dose chemotherapy in patients with poor-prognosis breast cancer.


British Journal of Cancer (1998) 78(7), 913–921
© 1998 Cancer Research Campaign

### High-dose chemotherapy followed by reinfusion of selected CD34+ peripheral blood cells in patients with poor-prognosis breast cancer: a randomized multicentre study

C Chabannon[1], K Cornetta[2], J-P Lotz[3], C Rosenfeld[4], M Shlomchik[5], S Yanovitch[6], J-P Marolleau[7], G Sledge[2], G Novakovitch[1], EF Srour[2], B Burtness[5], J Camerlo[1], G Gravis[1], J Lee-Fischer[7], C Faucher[7], I Chabbert[7], D Krause[1], D Maraninchi[1], B Mills[8], L Kunkel[8], F Oldham[8], D Blaise[1] and P Viens[1]

[1]Institut Paoli-Calmettes. 232. boulevard Sainte Marguerite. 13273 Marseilles cédex 9. France: [2]Indiana University Medical Center. 550 North University Boulevard. UH 6611. Indianapolis. IN 46202. USA: [3]Hopital Tenon. 4. rue de la Chine. 75020 Paris cédex 20. France: [4]Texas Oncology PA. Medical City Dallas Hospital. 7777 Forest Lane Dallas. TX 75230. USA: [5]Yale-New Haven Hospital. Yale University School of Medicine. 330 Cedar Street. New Haven. CT 06510: [6]Virginia Commonwealth University North Hospital. 1300 East Marshall Street. PO Box 980230. Richmond. VA 23298: [7]Hospital Saint-Louis. 1 avenue Claude Vellefaux. 75475 Paris cédex 10. France: [8]Nexell Therapeutics Inc. 9. Parker. Irvine. CA 92618-1605. USA

**Summary** Seventy-one patients with poor-prognosis breast cancer were enrolled after informed consent in a multicentre randomized study to evaluate the use of selected peripheral blood CD34+ cells to support haematopoietic recovery following high-dose chemotherapy. Patients who responded to conventional chemotherapy were mobilized with chemotherapy (mainly high-dose cyclophosphamide) and/or recombinant human granulocyte colony-stimulating factor (rhG-CSF). Patients who reached the threshold of 20 CD34+ cells per μl of peripheral blood underwent apheresis and were randomized at that time to receive either unmanipulated mobilized blood cells or selected CD34+ cells. For patients in the study arm, CD34+ cells were selected from aphereses using the Isolex®300 device. Fifteen patients failed to mobilize peripheral blood progenitors and nine other patients were excluded for various reasons. Forty-seven eligible patients were randomized into two comparable groups. CD34+ cells were selected from aphereses in the study group. Haematopoietic recovery occurred at similar times in both groups. No side-effect related to the infusion of selected cells was observed. The frequency of epithelial tumour cells in aphereses was low (8 out of 42 evaluated patients), as determined by immunocytochemistry. We conclude that selected CD34+ cells safely support haematopoietic recovery following high-dose chemotherapy in patients with poor-prognosis breast cancer.

**Keywords:** breast cancer: haematopoietic stem cells: CD34: bone marrow transplantation: mobilized blood cell: tumour purging

Breast cancer is the most common malignancy affecting women in developed countries. Treatment efficacy and survival depend on a number of factors. Advanced breast cancer essentially remains an incurable disease. Combined surgery. radiotherapy and conventional chemotherapy regimens produce only 5–25% complete remissions and a median survival of 5–13 months in patients with metastatic breast cancer (Harris et al. 1997). There is an increasing interest in high-dose chemotherapy for patients with advanced breast cancer. Although definitive answers will await termination and analysis of large randomized European and North American trials. there are indications that increasing the dose intensity may contribute to a better outcome. at least for some subsets of patients with breast cancer (Bonadonna and Valagussa. 1981: Tannock et al. 1988: Dunphy et al. 1990: Wallerstein et al. 1990: Engelsman et al. 1991: Kennedy et al. 1991: Antman et al. 1992: Peters et al. 1993: Wood et al. 1994: Beswoda et al. 1996).

*Received 12 November 1997*
*Revised 25 February 1998*
*Accepted 5 March 1998*

*Correspondence to:* C Chabannon. Laboratoire de Biologie Cellulaire. Départment de Transfert de Gène et de Thérapie Génique. Institut Paoli-Calmettes. 232. boulevard Sainte Marguerite. 13273 Marseille cédex 9. France

Within the last few years. autologous mobilized peripheral blood cells have been substituted for autologous bone marrow. to provide haematopoietic support after high-dose chemotherapy for a variety of malignancies. including breast cancer (Gale et al. 1993: Gratwohl et al. 1996). Mobilized peripheral blood cells provide more rapid recovery of neutrophils and platelets than bone marrow after high-dose chemotherapy. thus reducing the morbidity. the use of medical resources and the cost of transplant procedures (Faucher et al. 1994: Hartmann et al. 1996: Schmitz et al. 1996: To et al. 1997). However. unmanipulated aphereses contain only a small proportion of progenitors. along with subsets of differentiated and accessory cells. and. in some cases. tumour cells (Ross et al. 1993: Brugger et al. 1994*a*): the latter appear to be mobilized by chemotherapy and haematopoietic growth factors to some extent (Brugger et al. 1994*a*). Because gene marking studies suggest that residual tumour cells in the graft may contribute to relapses occurring in a proportion of patients with other malignancies (Brenner et al. 1993: Deisseroth et al. 1994). there is an incentive to separate progenitors from other unnecessary cells present in the initial collection (Kennedy et al. 1991: Shpall et al. 1991*a*, *b*). Human haematopoietic progenitors express the CD34+ antigen (Andrews et al. 1992: Link et al. 1996: Bensinger et al. 1997). a sialomucin of unknown function (He et al. 1992: Simmons et al. 1992: Baumhueter et al. 1993). There is no clear demonstration so far that breast cancer cells express CD34. Therefore, selection of CD34+ cells may reduce or eliminate contaminating tumour cells from the graft. In addition to this 'purging' effect, CD34+ cell selection yields a well-characterized cell population – therefore producing a unique setting to study the relation between the number of infused progenitors and the haematopoietic recovery. Finally, selection of CD34+ cells leads to cryopreservation of small numbers of cells in a small volume, containing a small amount of cryoprotective agent, and may thus reduce both storage costs and side-effects associated with reinfusion of these cryoprotective agents such as dimethylsulphoxide (DMSO) (Shpall et al, 1997).

Engraftment after infusion of autologous selected CD34+ cells – either from bone marrow or mobilized blood cells – has already been reported (Berenson et al, 1991; Brugger et al, 1994b; Shpall et al, 1994; Gorin et al, 1995; Schiller et al, 1995; Civin et al, 1996; Lemoli et al, 1996; Mahé et al, 1996; Williams et al, 1996; Hohaus et al, 1997; Lopez et al, 1997; Mapara et al, 1997; Marin et al, 1997; McQuaker et al, 1997; Somlo et al, 1997). These studies – including patients with breast cancer – demonstrated that haematopoietic recovery occurred after infusion of selected CD34+ cells. However, with one exception (Shpall et al, 1997), a study based on the reinfusion of bone marrow cells, comparison with historical controls did not allow for the assessment of the influence of the selection process on the speed and durability of haematopoietic recovery, or of the incidence of side-effects. The present randomized multicentre study was designed to assess the safety and efficacy of using autologous selected CD34+ cells to support haematopoietic recovery following high-dose chemotherapy in patients with poor-prognosis breast cancer.

### PATIENTS AND METHODS

### Patients

From November 1994 to December 1995, 71 patients entered a randomized multicentre study designed to evaluate the safety and efficacy of selected peripheral blood CD34+ cells, to support autologous haematopoietic recovery after high-dose chemotherapy for poor-prognosis breast cancer. All patients had histologically documented adenocarcinoma of the breast; patients were considered to be at high-risk because they had metastatic disease, or because they had more than eight positive nodes, and were thus eligible for institutional protocols using dose-intensified chemotherapy with haematopoietic cell support. The study was reviewed and approved by the Comité Consultatif de Protection des Personnes dans la Recherche Biomédicale (CCPPRB) in Marseilles, for French centres, and by Institutional Review Boards at US institutions. All patients gave informed consent before entering the study. All patients were assigned a study number, but were not randomized until time of successful mobilization, as defined below.

### Collection of peripheral blood stem cells (PBSCs)

Peripheral blood progenitors were mobilized with a variety of regimens, according to institutional protocols: in most cases, it included high-dose cyclophosphamide (see Table 1), followed by the daily administration of recombinant human granulocyte colony-stimulating factor (rhG-CSF) (Neupogen, Amgen, Thousand Oaks, CA, USA) 5–10 μg kg$^{-1}$ or 300 μg subcutaneous, until collection of mobilized blood cells. At Indiana University, patients were mobilized with rhG-CSF alone (10 μg kg$^{-1}$ day$^{-1}$). Because a variety of mobilization regimens were used, a standard criteria for successful mobilization was established: apheresis was started when the absolute number of CD34+ cells in the peripheral blood rose above 20 μl.$^{-1}$ Patients who failed to reach this threshold were excluded. Patients who successfully mobilized were randomized at that time to the control arm or the test arm, using a computer-generated random assignment list for each participating medical centre. Randomized patients were further analysed on an intent-to-treat basis, unless otherwise indicated.

Aphereses were performed with an automated processor (CS3000®, Baxter-Fenwal Division, Deerfield, IL, USA, or Cobe Spectra®, Lakewood, CO, USA), and approximately two blood volumes were processed during each 3- to 4-h session. Aphereses were repeated on a daily basis until the equivalent of at least $2.5 \times 10^6$ CD34+ cells kg$^{-1}$ were collected for patients in the control arm, and at least $6.5 \times 10^6$ CD34+ cells kg$^{-1}$ were collected for patients in the study arm (of which $5 \times 10^6$ CD34+ cells kg$^{-1}$ were used for cell separation and $1.5 \times 10^6$ CD34+ cells kg$^{-1}$ were cryopreserved unmanipulated as a back-up). Patients who failed to reach these thresholds were removed from the study. These figures were based on reports that were available at initiation of the study, regarding the minimal number of CD34+ cells that ensures optimal engrafment (Bender et al, 1992; Bensinger et al, 1994; Haas et al, 1994; van de Wall et al, 1994; Faucher et al, 1996), and on the assumption that the yield of the cell separation procedure would be approximately 50%, thus providing for similar numbers of progenitors in both groups.

### Selection of CD34+ cells

CD34+ cells were separated from apheresis products, using the Isolex®300 device according to the manufacturer's instructions, with chymopapain as an agent to release immunomagnetic beads from target cells (Civin et al, 1996; Hohaus et al, 1997; Mapara et al, 1997). When CD34+ cells had to be separated on the day following apheresis, cells were stored overnight at 4°C or at room temperature. In brief, the cells were centrifuged at low speed to deplete platelets, and washed once with a buffer solution containing 1% (v/v) human serum albumin. The volume was adjusted to 100 ml, and 0.5 g of human immunoglobulin was added. Clinical grade murine class II anti-CD34 antibody (9C5) was also added to a final concentration of 0.5 μg $10^{-6}$ nucleated cells. After a 15-min incubation, the cells were washed once with a buffer solution, and transferred to the Isolex® disposable chamber, and the volume adjusted to 300 ml. An additional 0.5 g of human immunoglobulin and one vial of prewashed sheep anti-mouse IgG-coated paramagnetic beads (Dynabeads® M-450, Dynal) was added to the chamber. Following 30 min of incubation, the non-target (CD34 'minus') cells were drained from the chamber, and the target CD34+ cell–bead complexes were washed three times with a buffer solution. The cells were released from the beads by the addition of 8000 pkat clinical grade chymopapain (Chymo-Cell-T), which cleaves a chymopapain-sensitive epitope recognized by the 9C5 monoclonal antibody on the CD34 molecule (Greaves et al, 1995), followed by a 15-min incubation. The CD34+ cells – free of beads – were drained from the chamber and washed before cryopreservation.

Selected and unselected cell products were cryopreserved according to institutional procedures with 10% DMSO, using controlled rate freezing, with the exception of Texas Oncology PA in

 © Cancer Research Campaign 1998

**Table 1** Patient care and accrual

| Centre | Mobilization | Conditioning regimen | Patients |
|---|---|---|---|
| Marseille Institut Paoli-Calmettes | Cyclophosphamide 4 g $m^{-2}$<br>Doxorubicin 75 mg $m^{-2}$<br>rhG-CSF 300 μg $day^{-1}$ | Cyclophosphamide 120 mg $kg^{-1}$<br>Mitoxantrone 36 mg $m^{-2}$<br>Melphalan 140 mg/$m^{-2}$ | 27 |
| Indianapolis. Indiana University Medical Center | rhG-CSF 10 μg $kg^{-1}$ | Cyclophosphamide 6 g $m^{-2}$<br>Carboplatin 2 g $m^{-2}$<br>Etoposide 625 mg $m^{-2}$ | 24 |
| Paris. Hopital Tenon | Cyclophosphamide 3 g $m^{-2}$<br>Epirubicin 100 mg $m^{-2}$<br>rhG-CSF 300 μg $day^{-1}$ | Cyclophosphamide 120 mg $kg^{-1}$<br>Mitoxantrone 45 mg $m^{-2}$<br>Melphalan 140 mg $m^{-2}$ | 6 |
| Dallas. Texas Oncology PA | Cyclophosphamide 4 g $m^{-2}$<br>rhG-CSF 5 μg $kg^{-1}$ $day^{-1}$ | Cyclophosphamide 6 g $m^{-2}$<br>Thiotepa 500 mg $m^{-2}$<br>Carboplatin 800 mg $m^{-2}$ | 7 |
| New Haven. Yale University | Cyclophosphamide 5 g $m^{-2}$<br>rhG-CSF 5 μg $kg^{-1}$ $day^{-1}$ | Thiotepa 900 mg $m^{-2}$ | 5[a] |
| Richmond. Virginia Commonwealth University | Ifosfamide 4 g $m^{-2}$<br>Cisplatin 50 mg $m^{-2}$<br>Etoposide 500 mg $m^{-2}$<br>rhG-CSF 5 μg $kg^{-1}$ $day^{-1}$ | Cyclophosphamide 6 g $m^{-2}$<br>Thiotepa 500 mg $m^{-2}$<br>Carboplatin 800 mg $m^{-2}$ | 2 |
| Total | | | 71 |

[a]Patients accrued at Yale were included in a double transplant programme (cycle 1 included melphalan 140 mg $m^{-2}$: see Patients and methods). Because a majority of patients was accrued at two of the six participating centres, 65% of the patients actually received one of two conditioning regimens (cyclophosphamide. melphalan and mitoxantrone. or cyclophosphamide. carboplatin and etoposide)

Dallas (this centre contributed seven patients: Table 1). where a pentastarch cryopreservation solution and dump freezing were used.

The percentages of CD34⁺ cells and other cell subsets were estimated before and after the separation. using immunofluorescence and flow cytometry. according to institutional protocols. HPCA-2 (Becton Dickinson. San Jose. CA. USA). a class III anti-CD34 monoclonal antibody that recognizes a chymopapain-resistant epitope (Greaves et al. 1995) was used to measure the percentage of CD34⁺ cells. In order to assess the institutional variation in analysis of cell products for content of CD34⁺ cells. available immunofluorescence data from each site were provided on disks (no cell sample was sent) to a central lab (Hematologics. Seattle. WA. USA) for reanalysis. The percentage of CD34⁺ cells reported from the sites was compared with the percentage of CD34⁺ cells reported by the central analysis lab. There was no difference between the central lab and the individual labs as a group. when examining the percentage of CD34⁺ cells collected in apheresis products or in the CD34⁺ cell selection products. When compared individually with the central lab. only one group appeared to provide significantly different results ($P = 0.034$) for the percentage of CD34⁺ cells in apheresis products. and the difference was estimated to be 0.04% (although this difference was statistically significant. it appears to be clinically meaningless). The comparison of CD34⁺ cell numerations from different centres gives information on how similarly investigators interpreted flow cytometry data at different workplaces: it does not give information on how similarly they stain cells and acquire flow cytometry data. However. by demonstrating that all participating centres interpretated flow cytometry data very similarly. this is a good indication that all steps necessary for CD34⁺ cell measurements were probably performed in a similar manner. The percentage of B and T cells in cell products was also estimated. with monoclonal antibodies recognizing CD19 and CD3 respectively.

### Tumour cell contamination

The presence of epithelial tumour cells in aphereses and after CD34⁺ cell selection was independently assessed for all centres at BIS Laboratories (Reseda. CA. USA) (Moss and Ross. 1992: Ross et al. 1993). using cryopreserved cell samples that were collected at and sent from each participating institution. Cytospin slides were prepared. and at least $10^6$ cells were examined. Tumour cells were detected and quantified using immunocytochemistry (APAAP). and a combination of five different monoclonal antibodies: IgG1 antibodies 9184. 9187 and 9189 (Baxter Healthcare Corporation) recognize 200-kDa (c-erb2). 55-kDa and 42-kDa glycoproteins. respectively. which are specifically expressed on epithelial cells (Ross et al. 1993): SB3 (BIS Laboratories) recognizes 8-. 18- and 19-kDa cytokeratins and TFS-2 recognizes a cellular adhesion molecule. A breast cancer cell line was used as a positive control.

### Transplantation

Patients received a variety of conditioning regimens. most of them containing alkylating agents (Frei et al. 1989) and anthracyclines. according to institutional protocols (Table 1). At least 24 h after completion of the chemotherapy. patients received either cryopreserved unseparated autologous mobilized blood cells or cryopreserved selected CD34⁺ cells that were thawed at the bedside. immediately before infusion. Patients received rhG-CSF. 5 μg $kg^{-1}$ or 300 μg daily. starting at day 1 following transplantation. until

© Cancer Research Campaign 1998

**Table 2** Patient characteristics in non-randomized and treatment groups (for five patients accrued at Yale University Hospital, collection was planned for two transplants)

| | Non-randomized ($n$ = 24) | Control ($n$ = 21) | Test ($n$ = 26) |
|---|---|---|---|
| Median age (years) | 48 | 46 | 44.5 |
| Range | 34–64 | 31–62 | 28–60 |
| Stage | | | |
| II/III | 5 | 6 | 6 |
| IV | 21 | 12 | 14 |
| Metastatic sites | | | |
| Soft tissues | 15 | 3 | 4 |
| Bone | 10 | 10 | 10 |
| Marrow | 1 | 2 | 3 |
| Visceral* | 9 | 9 | 10 |
| More than one site | 8 | 6 | 8 |
| No. of previous regimens | | | |
| 1–2 | 50.0% | 57.2% | 69.2% |
| 3 | 22.7% | 33.3% | 15.4% |
| ≥ 4 | 27.3% | 9.5% | 15.4% |
| Prior radiotherapy | | | |
| None | 16 | 11 | 10 |
| Local | 3 | 10 | 16 |
| Mobilization regimens (No. of rhG-CSF/no. of chemo + rhG-CSF) | 59% (13/8) | 29% (6/15) | 27% (7/19) |
| No. of aphereses | NA | 2.2 (1–6) | 3.2 (2–5) |

**Table 3** Device performance summary and cell product characteristics

| | Control | | Test | |
|---|---|---|---|---|
| | Range ($n$) | Median | Range ($n$) | Median |
| *Apheresis product* | | | | |
| Total nucleated cells (× $10^9$) | 2.2–99.4 (44) | 12.1 | 1.4–50.3 (79) | 18.4 |
| % $CD34^+$ cells | 0.1–11.38 (47) | 0.78 | 0.2–9.1 (81) | 0.72 |
| % Viability | 90–100 (45) | 99 | 90–100 (77) | 99 |
| *Isolex® selected product* | | | | |
| Total nucleated cells (× $10^9$) | NA | NA | 0.02–0.95 (48) | 0.07 |
| % $CD34^+$ cells | NA | NA | 55–99 (48) | 89 |
| % Viability | NA | NA | 84–100 (48) | 99 |
| Yield $CD34^+$ cells (%) | NA | NA | 16–170 (48) | 44 |
| Log depletion $CD3^+$ cells | NA | NA | 2.2–4.6 (29) | 3.5 |
| Log depletion $CD19^+$ cells | NA | NA | 1.0–3.0 (21) | 2.3 |

neutrophil recovery (ANC > 0.5 × $10^9$ $l^{-1}$ for at least 2 consecutive days). Supportive care (transfusions, antibiotics, etc.) was provided according to institutional protocols.

### Assay for human anti-mouse antibodies (HAMA) and human anti-sheep antibodies (HASA)

To test for HAMA, the test system was an ELISA assay for human antibody to murine IgG using the ImmuSTRIP® HAMA IgG test (Immunomedics, Warren, NJ, USA). There is neither a reference method nor reference material for HAMA testing; therefore a known amount of standard was added to human serum. The difference between the HAMA concentration before and after 'spiking' was expressed as a per cent recovery of the amount added. Patient results were reported as falling into one of the following three categories: negative (< 74 ng $ml^{-1}$), positive (74–440 ng $ml^{-1}$) or strongly positive (> 440 ng $ml^{-1}$).

A similar ELISA assay was used to determine whether HASA was present. A ratio $R$ was established between the sample and a normal control. The samples were scored as follows: if the sample was $1.0 < R < 2.5$, the sample was considered negative; if $2.5 < R < 7.5$, the sample was considered positive; $7.5 < R < 10.0$ was classified as double positive; $10.0 < R < 20.0$ was classified as triple positive.

### Statistical analyses

Statistical analyses were carried out, using the SAS statistical software package. All statistical tests were two-sided, and a significance level of 0.05 was used to judge statistically significant test results. The primary efficacy objective was to determine whether infusion of $CD34^+$ cells isolated from mobilized blood cells results in any delay of neutrophil engraftment (delay being defined as more than 3 days), relative to neutrophil engraftment following transplant with unselected mobilized blood cells. Kaplan–Meier estimates of time to neutrophil recovery were calculated for the control group (unselected mobilized blood cells) and the test group (selected $CD34^+$ mobilized blood cells), and the log-rank test was used to assess treatment differences. A sample size of 20 patients per treatment arm provides 81.5% power to detect a 3-day difference in median time to neutrophil engraftment, with a level of significance of 0.056. Secondary engraftment parameters included time to neutrophils above 1 × $10^9$ $l^{-1}$, and times to platelets above 20 × $10^9$ $l^{-1}$, 50 × $10^9$ $l^{-1}$ and 100 × $10^9$ $l^{-1}$.

The primary safety objective was to determine whether infusion of $CD34^+$ isolated from mobilized blood cells results in any unusual or unexpected toxic side-effect over those observed after infusion of unselected mobilized blood cells. The incidence of serious and unexpected side-effects was calculated for each treatment group, and Fisher's exact test used to assess treatment differences. The incidence of infectious episodes with or without documentation (culture) was calculated for each treatment group. Secondary safety parameters included days of hospitalization following chemotherapy, days of antibiotic usage and number of transfusions required for each group.

### RESULTS

### Patient characteristics and blood cell mobilization

A total of 71 patients were entered in the study from November 1994 to December 1995, at six different medical centres, using the Isolex®300 device to separate $CD34^+$ cells from aphereses. Of the 71, 24 patients were not randomized, 15 because they failed to meet the mobilization criteria (the number of circulating $CD34^+$ cells did not reach 20 $\mu l^{-1}$), and nine because of a variety of events (two patients had progressive disease, three patients had medical complications before randomization, one patient was assigned a number in error, one patient voluntarily withdrew from the study before randomization and two patients were not monitored for mobilization). The other 47 patients reached the threshold of 20 $CD34^+$ cells $\mu l^{-1}$ of circulating blood, and were randomly assigned either to the test group ($n$ = 26), or to the control group ($n$ = 21).

© Cancer Research Campaign 1998

**Table 4** Engraftment summary in the control and test groups

| | CD34+ cell dose (× $10^6$ kg)$^{-1}$ | | ANC > 500 ml$^{-1}$ (days) | | ANC > 1000 ml$^{-1}$ (days) | | Platelets > 20 000 ml$^{-1}$ (days) | | Platelets > 50 000 ml$^{-1}$ (days) | | Platelets > 100 000 ml$^{-1}$ (days) | |
|---|---|---|---|---|---|---|---|---|---|---|---|---|
| | Range | Median | Range | Median | Range | Median | Range | Median | Range | Median | Range | Median |
| Control $n$ = 21 | 2.7–40.1 | 4.4 | 8–14 | 10.0 | 8–21 | 11 | 6–60 | 10.0 | 8–88 | 14.0 | 10–198 | 20 |
| Test $n$ = 26 | 0.8–16.0 | 2.7 | 9–15 | 11.0 | 9–19 | 12 | 8–40 | 12.0 | 11–40 | 17.5 | 14–174 | 28 |
| Test subset[a] ($n$ = 21) | 0.8–16.0 | 2.3 | 9–14 | 11.0 | 9–14 | 12 | 9–40 | 12.0 | 13–40 | 17.0 | 14–83 | 28 |

[a]Patients in the test group who did not receive a back-up.

Following randomization, ten additional patients did not complete the study as initially planned, for reasons including unsatisfactory apheresis collections, voluntary withdrawal and death. However, the analysis was carried out on an intent-to-treat basis, and includes these patients, except where indicated.

Tables 1 and 2 describe characteristics of non-randomized and randomized patients. There was a slight but not significant excess of patients who had received three or more chemotherapy regimens before study inclusion in the non-randomized group. There was also an excess of patients who were mobilized with rhG-CSF alone in this group. The control and test groups were comparable in terms of staging, sites of metastases, prior treatments (including chemotherapy, hormone therapy, radiation therapy and surgery) and centre of origin. It is noticeable that, whereas 43.5% of the patients had bone metastases, only 10.1% had documented marrow involvement.

### Cell selection: progenitor cell recovery and tumour purging

Table 3 shows the characteristics of collected products in both groups. Following selection with the Isolex®300 device, the median recovery of CD34⁺ cells was 44%, and the median purity of CD34⁺ cells was 89%. The selection resulted in a final product that contained a median number of 75 × $10^6$ nucleated cells, representing a greater than 100-fold reduction from the starting number of cells in the apheresis product. Despite the fact that many patients had two or more separations, the number of CD34⁺ cells that were cryopreserved for patients in the test group was 2.7 × $10^6$ CD34⁺ cells kg$^{-1}$ (2.3 × $10^6$ CD34⁺ cells kg$^{-1}$ in the subset of patients who did not receive a back-up), a value that is significantly lower than for the control group (4.4 × $10^6$ CD34⁺ cells kg$^{-1}$; $P$ = 0.0024; Table 4).

**Table 5** Patients and products tested for the presence of breast tumour cells by BIS Laboratories using immunocytochemistry (see Patients and methods)

| | Selected CD34 | Unselected PBSCs |
|---|---|---|
| No. of patients tested | 24 | 18 |
| No. negative | 19 | 15 |
| No. of positive apheresis products | 5 | 3 |
| No. of positive CD34 products | 2 | NA |
| No. of CD34 products ND or NE | 3 | NA |

ND, not done; NE, not evaluable.

Table 5 describes the detection of epithelial tumour cells in aphereses. The overall frequency of positive products was 12 out of 84 aphereses, or 14.3%, and 8 out of 42 tested patients, or 19%. In positive aphereses, the frequency of epithelial cells ranged from 1 in 2 × $10^6$ to 3 in $10^5$ cells. In 5 out of the 8 positive patients,

**Figure 1** **A** Kaplan–Meier analysis of time to ANC recovery (ANC > 0.5 × $10^9$ l$^{-1}$). **B** Kaplan–Meier analysis of time to platelet recovery (platelets > 20 × $10^9$ l$^{-1}$) (—, control group; - - -, test group)

**Table 6** Description of clinical events and transfusion requirements in the control group and the test group

| | Control | | | Test | | | P |
|---|---|---|---|---|---|---|---|
| Variable | n | Mean | Range | n | Mean | Range | |
| Random donor platelet units | 21 | 4.2 | 0–28 | 26 | 2.7 | 0–35 | 0.4513 |
| Single donor platelet units | 21 | 4.4 | 0–18 | 26 | 4.0 | 0–29 | 0.8733 |
| RBC units | 21 | 5.10 | 2–18 | 26 | 5.54 | 2–25 | 0.6704 |
| Days in hospital | 20 | 25.9 | 17–100 | 26 | 23.0 | 11–48 | 0.5598 |

**Table 7** Distribution of infectious episodes

| | Infectious disease | | | |
|---|---|---|---|---|
| | No | | Yes | |
| | n | % | n | % |
| Control group | 9 | 42.86 | 12 | 57.14 |
| Test group | 12 | 46.15 | 14 | 53.85 |
| Total | 21 | 44.68 | 26 | 55.32 |

consecutive aphereses were not all positive. Five patients in the study group had positive aphereses, but only two of these had adequate samples for assay of the CD34⁺ cell product, and therefore offered an opportunity to assess the results of CD34⁺ cell selection in terms of tumour purging: in both cases, immunocytochemistry still revealed the presence of epithelial cells in the final product, with a frequency of 1–2 in $10^5$ cells. Because the number of positively stained cells reported for each sample is so small, only rough calculations of log tumour cell reductions can be made, taking into account the reduction in the absolute number of cells in the final product: this resulted in an approximately 2.5 log decrease in the number of tumour cells that were reinfused to the patient, when compared with unseparated PBSCs. Based on the total number of nucleated cells in the final selected product, this represents the reinfusion of 100 to 1000 tumour cells in patients in the test group, who were positive before selection.

$CD34^+$ cell selection also resulted in a decrease in other non-target cell populations, including T lymphocytes ($CD3^+$ cells, 3.5 logs) and B lymphocytes ($CD19^+$ cells, 2.3 logs).

### High-dose chemotherapy and engraftment

Patients received high-dose chemotherapy according to institutional protocols (Table 1), followed by reinfusion of thawed cell products: these contained a significantly lower number of $CD34^+$ cells for patients in the test group than patients in the control group ($P$ = 0.0024; Table 4). A total of 5 out of 26 patients in the test group received the back-up (four patients were target collection failures with less than $5 \times 10^6$ $CD34^+$ cells $kg^{-1}$, and the dose of progenitor cells following selection was considered insufficient for the fifth one), but are analysed on an intent-to-treat basis, except where indicated. Figure 1A and B shows the cumulative probability (Kaplan–Meier plot) of achieving an ANC above $0.5 \times 10^9$ $l^{-1}$ and a platelet number above $20 \times 10^9$ $l^{-1}$. There was no significant difference between the two groups in time to neutrophil or platelet recovery. Thus, one can be confident that the test arm engrafts neutrophils and platelets no more than 3 days later than the control group. Comparison of transfusion requirements, days of antibiotics use and days of hospitalization did not show any significant difference between the control and test groups (Table 6). To eliminate the possibility that the five patients who received a back-up product (unselected apheresis cell products) in the test group may have biased the engraftment data, a subset of the test group including the 21 patients who received only $CD34^+$ cells was analysed. Conclusions remained unchanged, with no difference between the subset and the control group in terms of neutrophil or platelet engraftment. There has been no report of long-term marrow dysfunction.

There was no difference between the test group and the control group in the reported incidence of any adverse effect associated with infusion (reported according to the NCI common toxicity criteria). The number of infectious episodes was identical in both groups, with 57% in the control group and 54% in the test group (Table 7); there was also no difference in the severity of infectious episodes: this is supported by comparable antibiotic usage in both groups. One patient in the control group had two episodes of fungal infections, and one episode of viral infection: no other documented opportunistic infection was reported. No infusional toxicity in the test group was considered to have any relationship with the use of the cell selection device. Analysis of variance to compare the maximal changes in vital signs (respiration, pulse, temperature, diastolic and systolic blood pressure) from before infusion to after infusion indicated no difference between the two groups. Serum samples from 11 patients in the test group and 11 patients in the control group were checked for the presence of HAMA and HASA, and were found to be negative at baseline and after transplantation, except for one control patient who was positive for HASA at both time points.

Thirteen of 26 test patients and 13 of 21 control patients have been reported to have relapsed. There have been four reported deaths of patients during the first 6 months after transplant, three in the test group and one in the control group. All had progressive disease. There have been eight additional deaths reported in the long-term follow-up, four in each group. However, it should be noted that two of the relapses and deaths in the test arm occurred in patients who received back-up unselected products. In order to better assess the potential effect of positive $CD34^+$ cell selection on relapse and death, we have reanalysed these parameters, comparing the control group with the test subset, excluding the five patients who received unselected back-up PBSCs: this did not change conclusions. At more than 1 year of follow-up for most patients (median follow-up of 344 days), there is no significant difference in terms of event-free or overall survival between the control and test groups, and no pattern suggestive that the test group is at greater risk.

© Cancer Research Campaign 1998

### DISCUSSION

Here we demonstrate in a prospective randomized study that selected autologous peripheral blood CD34+ cells support haematopoietic recovery following high-dose chemotherapy in patients with poor-prognosis breast cancer, in a similar manner to unselected mobilized peripheral blood cells. Times to neutrophil and platelet recovery were unaffected by the cell selection in a group of 47 randomized patients who adequately mobilized blood progenitors as assayed by a number of circulating CD34+ cells above 20 $\mu l^{-1}$. There was a trend to increased time to platelet recovery, an observation similar to a previously published report (Somlo et al, 1997), but this was not statistically significant, nor did it translate into more adverse events, or additional requirements for platelet transfusion. These observations were obtained in a heterogeneous cohort of subjects with poor-prognosis breast cancer, which reflects, however, the reality of medical practice in this field, and the lack of standards of care for these patients. In addition, characteristics of patients were equally distributed in the test group and in the control group, and the study design – including randomization stratified by participating centre – allowed for the power to detect a 3-day difference in the time to neutrophil engraftment.

It is clear that CD34+ cell selection results in a significant loss of progenitors. Whereas the study was initially designed so that patients in the study group and patients in the control group should have received comparable numbers of CD34+ cells, analysis of infused cell counts showed that this was not the case. The 26 patients in the study group received a significantly lower number of CD34+ cells than did the 21 patients in the control group, despite a significantly higher number of aphereses in the former. These observations reflect both the difficulty during apheresis of collecting accurately a predefined number of cells (e.g. more than $2.5 \times 10^6$ CD34+ cells $kg^{-1}$ were collected for most control patients), and the loss of cells during the separation procedure. It is interesting that the number of CD34+ cells that were infused in patients in the study group was below the threshold that we (Faucher et al, 1996) and others (Bender et al 1992; Bensinger et al, 1994; Haas et al, 1994; Weaver et al 1995) considered as the minimal number necessary to ensure the quickest haematological recovery with unselected PBSCs; on the other hand, patients in the control group received on average a number of CD34+ cells that was close to or above this threshold. The loss of progenitor cells may explain the delay in platelet recovery observed after transplantation of autologous marrow CD34+ cells, which was previously demonstrated (Shpall et al, 1997). It is also clear from our observations that before cell selection one has to use the best mobilization regimen that is available: because chemotherapy and haematopoietic growth factors usually result in a better mobilization than haematopoietic growth factors alone, the former may be the preferred choice for this kind of treatment strategy; indeed, there was an excess of patients mobilized with rhG-CSF alone in the group of patients who failed to meet the mobilization criteria compared with the group of randomized patients. The use of rhG-CSF alone in a previously published study (Mahé et al, 1996) resulted in an average number of infused CD34+ cells that was lower than in the present study, especially for the subset of myeloma and lymphoma patients who had already received more than three courses of chemotherapy. Finally, patients who have a medical history that predicts a poor ability to mobilize blood progenitors – such as a high number of previous chemotherapy cycles (Bensinger et al, 1994; Haas et al, 1994; Chabannon et al, 1995; Mahé et al, 1996) – may not be good candidates for cell selection, unless the yield of CD34+ cells can be improved; although the difference was not statistically significant, there were more patients who had received three or more chemotherapy regimens before inclusion in the group of patients who failed to reach the threshold of 20 circulating CD34+ cells $\mu l^{-1}$ of blood (non-randomized patients).

The feasibility of CD34+ selection has already been demonstrated, both for patients with breast cancer and for patients with other malignancies (Berenson et al, 1991; Brugger et al, 1994b; Shpall et al, 1994; Gorin et al, 1995; Schiller et al, 1995; Civin et al, 1996; Lemoli et al, 1996; Mahé et al, 1996; Hohaus et al, 1997; Lopez et al, 1997; Mapara et al, 1997; Marin et al, 1997; McQuaker et al, 1997; Somlo et al, 1997). The most recent studies describe the use of apheresis products (Brugger et al, 1994*b*; Schiller et al, 1995; Lemoli et al, 1996; Lopez et al, 1997; Mahé et al, 1996; Hohaus et al, 1997; Mapara et al, 1997; Marin et al, 1997; Somlo et al, 1997), whereas older publications are based on the selection of CD34+ cells from marrow collections (Berenson et al, 1991; Shpall et al, 1994; Gorin et al, 1995; Civin et al, 1996; Lopez et al, 1997; Shpall et al, 1997). Also, only three publications (Civin et al, 1996; Hohaus et al, 1997; Mapara et al, 1997) describe the use of a biomedical device similar to the one used in this study. Except for one study with marrow grafts (Shpall et al, 1997) none of these studies was a randomized study, and haematological recovery was compared with historical controls. We here provide additional evidence that CD34+ cells can be safely selected from apheresis products. Because aphereses contain a higher number of haematopoietic progenitors than marrow harvests, it is likely that the consequences of the loss of CD34+ cells during the procedure will have less influence on autologous haematopoietic recovery.

Tumour purging evaluation was difficult for several reasons. First, the frequency of positive aphereses is low (equal or lower than in the bone marrow), even in a population of patients treated at an advanced stage of their disease; the significance of this low frequency, and the most appropriate technique to be used for tumour cell detection in breast cancer remain to be determined (Mapara et al, 1997). Our observations consistently demonstrate the possibility of detecting as few as one epithelial tumour cell in $10^5$ to $10^6$ assayed cells, and the percentage of positive aphereses and patients in our series is consistent with data already published (Ross et al, 1993; Brugger et al, 1994*a*; Jones, 1994; Hohaus et al, 1997); only one recent report suggests a much higher frequency of positive aphereses in advanced breast cancer patients (Mapara et al, 1997). Second, the clinical consequences of tumour purging are difficult to assess on such a small number of subjects, and are unlikely to be apparent in a population of patients who mostly had advanced metastatic disease. In two evaluable patients, detection of epithelial tumour cells among progenitors, before and after separation, showed that purging was considerable, albeit partial, resulting in the reinfusion of a very small number of tumour cells; this small number is to be compared with the high tumour burden that characterized all of our patients at the time of high-dose chemotherapy. The observation that detection of bone marrow micrometastases using PCR is a strong adverse prognostic factor (Fields et al, 1996) suggests that eradication of the endogenous disease remains the most important hurdle in the area of metastatic breast cancer chemotherapy. The clinical consequences and potential advantages of tumour purging will now need to be evaluated in larger cohorts of patients, and are likely to be more apparent in a

© Cancer Research Campaign 1998

population of subjects that are transplanted with a lower tumour burden, such as in the adjuvant setting. However, because the prognosis of this patient population is much better than for patients with metastatic disease, it is likely that this evaluation will require a large number of patients and long-term follow-up.

Analysis of the outcome of patients in both groups showed no difference in terms of relapse rate and event-free survival. As could be expected from the characteristics of this group of patients, relapse occurred frequently and early after transplantation, and progressive disease accounted for all recorded deaths. This emphasizes the need for better chemotherapy regimens in patients with poor-prognosis breast cancer (the present study was however designed to evaluate a new transplant technique, and not the use of high-dose chemotherapy for the treatment of advanced or poor-prognosis breast cancer), and again the need to choose a different population of patients to evaluate the consequences of tumour purging, once the safety of using selected blood $CD34^+$ cells is definitively established.

In conclusion, we were able to select safely $CD34^+$ cells from aphereses collected in patients with poor-prognosis breast cancer. Our randomized study demonstrates that selected $CD34^+$ cells support haematopoietic recovery after high-dose chemotherapy in these patients, in a time period similar to that observed in patients receiving unmanipulated autologous mobilized blood cells, despite the loss of progenitors during ex vivo cell processing, but thus at the expense of one additional apheresis. As the demonstrable clinical benefits associated with positive selection may be relatively small compared with the many risks associated with high-dose chemotherapy and progenitor cell reinfusion, it was important to demonstrate that the risks associated with the use of a cell selection device are relatively small. Our observations prepare the way for future trials designed to evaluate the clinical benefits associated with $CD34^+$ cell selection.

### REFERENCES

Andrews RG, Bryant AM, Bartelmez SH, Muirhead DY, Knitter GH, Bensinger W, Strong DM and Bernstein ID (1992) CD34+ marrow cells devoid of T and B lymphocytes reconstitute stable lymphopoiesis and myelopoiesis in lethally irradiated allogeneic baboons. *Blood* **80**: 1693–1701

Antman K, Ayash L, Elias A, Wheeler C, Hunt M, Eder JP, Teicher BA, Critchlow J, Bibbo J, Schnipper LE and Frei E (1992) A phase II study of high-dose cyclophosphamide, thiotepa, and carboplatin with autologous marrow support in women with measurable advanced breast cancer responding to standard-dose therapy. *J Clin Oncol* **10**: 102–110

Baumhueter S, Singer MS, Henzel W, Hemmerich S, Renz M, Rosen SD and Lasky, LA (1993) Binding of L-selectin to the vascular sialomucin CD34. *Science* **262**: 436–438

Bender JG, To LB, Williams S and Schwartzberg LS (1992) Defining a therapeutic dose of peripheral blood stem cells. *J Hematother* **1**: 329–341

Bensinger WI, Longin K, Appelbaum F, Rowley S, Weaver C, Lilleby K, Gooley T, Lynch M, Higano T, Klarnet J, Chauncey T, Storb R and Buckner CD (1994) Peripheral blood stem cells (PBSCs) collected after recombinant granulocyte colony stimulating factor (rhG-CSF): an analysis of factors correlating with the tempo of engraftment after transplantation. *Br J Haematol* **87**: 825–831

Bensinger WI, Buckner CD, Shannon-Dorcy K, Rowley S, Appelbaum FR, Benyunes M, Clift R, Martin P, Demirer T, Storb R, Lee M and Schiller G (1997) Transplantation of allogeneic CD34+ peripheral blood stem cells in patients with advanced hematologic malignancy. *Blood* **88**: 4132–4138

Berenson RJ, Bensinger WI, Hill RS, Andrews RG, Garcia-Lopez J, Kalamasz DF, Still BS, Spitzer G, Buckner CD, Bernstein ID and Thomas ED (1991) Engraftment after infusion of CD34+ marrow cells in patients with breast cancer or neuroblastoma. *Blood* **77**: 1717–1722

Beswoda WR, Seymour L and Dansey RD (1996) High-dose chemotherapy with hematopoietic rescue as primary treatment for metastatic breast cancer: a randomized trial. *J Clin Oncol* **13**: 2483–2489

Bonadonna G and Valagussa P (1981) Dose–response effect of adjuvant chemotherapy in breast cancer. *N Engl J Med* **304**: 10–15

Brenner MK, Rill DR, Moen RC, Krance RA, Mirro J, Anderseon WF and Ihle JN (1993) Gene-marking to trace origin of relapse after autologous bone marrow transplantation. *Lancet* **341**: 85–86

Brugger W, Bross KJ, Glatt MG, Weber F, Mertelsmann R and Kanz L (1994*a*) Mobilization of tumor cells and hematopoietic progenitor cells into peripheral blood of patients with solid tumors. *Blood* **83**: 636–640

Brugger W, Henschler R, Heimfeld S, Berenson RJ, Mertelsmann R and Kanz L (1994*b*) Positively selected autologous blood CD34+ cells and unseparated peripheral blood progenitor cells mediate identical hematopoietic engraftment after high dose VP16, ifosfamide, carboplatin, and epirubicin. *Blood* **84**: 1421–1426

Chabannon C, Le Coroller AG, Faucher C, Novakovitch G, Blaise D, Moatti JP, Maraninchi D and Mannoni P (1995) Patient condition affects the collection of peripheral blood progenitors after priming with recombinant granulocyte colony-stimulating factor. *J Hematother* **4**: 171–179

Civin CI, Trischmann T, Kadan NS, Davis J, Noga S, Cohen K, Duffy B, Groenewegen I, Wiley J, Law P, Hardwick A, Oldham F and Gee A (1996) Highly purified CD34-positive cells reconstitute hematopoiesis. *J Clin Oncol* **14**: 2224–2233

Deisseroth AB, Zu Z, Claxton D, Hanania EG, Fu S, Ellerson D, Goldberg L, Thomas M, Janicek K, Anderson WF, Hester J, Korbling M, Durett A, Moen R, Berenson R, Heimfeld S, Hamer H, Calvert L, Tibbits P, Talpaz M, Kantarjian H, Champlin R and Reading R (1994) Genetic marking shows that Ph+ cells present in autologous transplants of chronic myelogenous leukemia (CML) contribute to relapse after autologous bone marrow in CML. *Blood* **83**: 3068–3076

Dunphy FR, Spitzer G, Breadar AU, Hortobagyi A, Horwitz LJ, Yau JC, Spinolo JA, S. J. F. H. Wallesrtein RO, Bohannan PA and Dicke KA (1990) Treatment of estrogen receptor negative or hormonally refractory breast cancer with double high-dose chemotherapy intensification and bone marrow support. *J Clin Oncol* **8**: 1207–1216

Engelman E, Klijn JCM, Rubens RD, Wildiers J, Beex LVAM, Nooij MA, Rotmensz N and Sylvester R (1991) Classical CMF versus a 3-weekly intravenous CMF schedule in post-menopausal patients with advanced breast cancer. *Eur J Cancer* **27**: 966–970

Faucher C, Le Coroller A-G, Blaise D, Novakovich G, Mannoni P, Moatti J-P and Maraninchi D (1994) Comparison of G-CSF primed peripheral blood progenitor and bone marrow autotransplantations: clinical assesment and cost-effectiveness study. *Bone Marrow Transpl* **14**: 895–901

Faucher C, Le Corroller AG, Chabannon C, Viens P, Stoppa AM, Bouabdallah R, Camerlo J, Vey N, Gravis G, Gastaut JA, Novakovitch G, Mannoni P, Bardou VJ, Moatti JP, Maraninchi D and Blaise D (1996) Autologous transplantation of blood stem cells mobilized with G-CSF alone in 93 patients with malignancies: the number of CD34+ cells reinfused is the only factor predicting both granulocyte and platelet recovery. *J Hematother* **5**: 663–670

Fields KK, Elfenbein GJ, Trudeau WL, Perkins JB, Janssen WE and Mascinski LC (1996) Clinical significance of bone marrow metastases as detected using the polymerase chain reaction in patients with breast cancer undergoing high-dose chemotherapy and autologous bone marrow transplantation. *J Clin Oncol* **14**: 1868–1876

Frei E, Antman K, Teicher B, Eder P and Schnipper L (1989) Bone marrow transplantation for solid tumours – prospects. *J Clin Oncol* **7**: 515–526

Gale RP, Butturini A and Henon PR (1993) Transplants of blood-derived hematopoietic cells. In *Peripheral Blood Stem Cell Autografts*, Wunder E and Henon P (eds), pp. 19–25. Springer-Verlag: Berlin-Heidelberg

Gorin NC, Lopez M, Laporte JP, Quittet P, Lesage S, Lemoine F, Berenson RJ, Isnard F, Grande M, Stachowiak J, Labopin M, Fouillard L, Morel P, Jouet JP, Noël-Walter MP, Detourmignies L, Aoudjhane M, Bauters F, Najman A and Douay L (1995) Preparation and successful engraftment of purified CD34+ bone marrow progenitor cells in patients with non-Hodgkin's lymphoma. *Blood* **86**: 1647–1654

Gratwohl A, Hermans J and Baldomero H (1996) Hematopoietic precursor cell transplants in Europe: activity in 1994. Report from the European Group for Blood and Marrow Transplantation. *Bone Marrow Transpl* **17**: 137–148

Greaves MF, Titley I, Colman SM, Bühring HJ, Campos L, Castoldi GL, Garrido F, Gaudernack G, Girard JP, Inglès-Esteve J, Invernizzi R, Knapp W, Lansdorp PM, Lanza F, Merle-Béral H, Parravicini C, Razak K, Ruiz-Cabello F, Springer TA, Van der Schoot CE and Sutherland DR (1995) CD34 cluster workshop report. In *Leukocyte Typing V, White Cell Differentiation Antigens*, Vol. 1, Schlossman SF, Boumsell L, Gilks W, Harlan JM, Kishimoto T, Morimoto C, Ritz J, Shaw S, Silverstein R, Springer T, Tedder TF and Todd RF (eds), pp. 840–846. Oxford University Press: Oxford

© Cancer Research Campaign 1998

Haas R. Möhle R. Frühauf S. Goldschmidt H. Witt B. Flentje M. Wannenmacher M and Hunstein W (1994) Patient characteristics associated with successful mobilizing and autografting of peripheral blood progenitor cells in malignant lymphoma. *Blood* **83**: 3787–3794

Harris JR. Morrow M and Norton L (1997) Malignant tumors of the breast. In *Cancer Principles and Practice of Oncology*. DeVita VT. Hellman S and Rosenberg SA (eds) pp. 1557–1616. JB Lippincott: Philadelphia

Hartmann O. Le Coroller AG. Blaise D. Michon J. Philip I. Norol F. Janvier M. Pico JL. Baranzelli MC. Rubie H. Coze C. Pinna A. Méresse V and Benhamou E (1996) Peripheral blood stem cells and bone marrow transplantation for solid tumors and lymphomas: hematologic recovery and costs. *Ann Int Med* **126**: 600–607

He XY. Antao VP. Basila D. Marx JC and Davis BR (1992) Isolation and molecular characterization of the human CD34 gene. *Blood* **79**: 2296–2302

Hohaus S. Pförsich M. Murea S. Abdallah A. Lin YS. Funk L. Voso MT. Kaul S. Schmid H. Wallwiener D and Haas R (1997) Immunomagnetic selection of CD34+ peripheral blood stem cells for autografting in patients with breast cancer. *Brit J Haematol* **97**: 881–888

Kennedy MJ. Beveridge RA. Rowley SD. Gordon GB. Abeloff MD and Davidson NE (1991) High-dose chemotherapy with reinfusion of purged autologous bone marrow following dose intensive induction as initial therapy for metastatic breast cancer. *J Natl Cancer Inst* **83**: 920–926

Lemoli RM. Fortuna A. Motta MR. Rizzi S. Giudice V. Nanetti A. Martinelli G. Cavo M. Amabile M. Mangianti S. Fogli M. Conte R and Tura S (1996) Concomitant mobilization of plasma cells and hematopoietic progenitors into peripheral blood of multiple myeloma patients: positive selection and transplantation of enriched CD34+ cells to remove circulating tumor cells. *Blood* **87**: 1625–1634

Link H. Arseniev L. Bähre O. Kadar JG. Diedrich H and Poliwoda H (1996) Transplantation of allogeneic CD34+ blood cells. *Blood* **87**: 4903–4909

Lopez M. Lemoine FM. Firat H. Fouillard L. Laporte JP. Lesage S. Isnard F. Stachowiak J. Ferre-Le Coeur F. Morel P. Najman A. Douay L and Gorin NC (1997) Bone marrow versus peripheral blood progenitor cells CD34 selection in patients with non-Hodgkin's lymphomas: different levels of tumor cell reduction. Implications for autografting. *Blood* **90**: 2830–2838

McQuaker IG. Haynes AP. Anderson S. Stainer C. Owen RG. Morgan GJ. Lumley M. Milligan D. Fletcher J. Bessell EM. Davis JM and Russell NH (1997) Engraftment and molecular monitoring of CD34+ peripheral blood stem cell transplants for follicular lymphoma: a pilot study. *J Clin Oncol* **15**: 2288–2295

Mahé B. Milpied N. Hermouet S. Robillard N. Moreau P. Letortorec S. Rapp MJ. Bataille R and Harousseau JL (1996) G-CSF alone mobilizes sufficient peripheral blood CD34+ cells for positive selection in newly diagnosed patients with myeloma and lymphoma. *Br J Haematol* **92**: 263–268

Mapara M. Körner IJ. Hildebrandt M. Bergou R. Krahl D. Reichardt P and Dörken B (1997). Monitoring of tumor cell purging after highly efficient immunomagnetic selection of CD34 cells from leukapheresis products in breast cancer patients: comparison of immunocytochemical tumor cell staining and reverse transcriptase-polymerase chain reaction. *Blood* **89**: 337–344

Marin GH. Dal Cortivo L. Cayuela JM. Matolleau JP. Pautier P. Jean-Zelek I. Brice P. Makke J. Benbunan M and Gisselbrecht C (1997) Peripheral blood stem cell CD34+ autologous transplant in relapsed follicular lymphoma. *Hematol Cell Ther* **39**: 33–40

Moss TJ and Ross AA (1992) The risk of tumor cell contamination in peripheral blood stem cell collections. *J Hematother* **1**: 225–232

Peters WP. Ross M. Vredenburgh JJ. Meisenberg B. Marks LB. Winer E. Kurzberg J. Bast Jr RC. Jones R. Shpall E. Wu K. Rosner G. Gilbert C. Mathias B. Coniglio D. Petros W. Craig Henderson I. Norton L. Weiss RB. Budman D and Hurd D (1993) High-dose chemotherapy and autologous bone marrow support as consolidation after standard-dose adjuvant therapy for high-risk primary breast cancer. *J Clin Oncol* **11**: 1132–1143

Ross AA. Cooper BW. Lazarus HM. Mackay W. Moss TJ. Ciobanu N. Tallman MS. Kennedy MJ. Davidson NE. Sweet D. Winter C. Adard L. Janse J. Copelan E. Meagher RC. Herzig RH. Klumpp TR. Kanh DG and Warner NE (1993) Detection and viability of tumor cells in peripheral blood stem cell collections from breast cancer patients using immunocytochemical and clonogenic assay techniques. *Blood* **82**: 2605–2610

Schiller G. Vescio R. Freytes C. Spitze G. Sahebi F. Lee M. Wu CH. Cao J. Lee JC. Ho G. CH. Lichtenstein A. Lill M. Hall J. Berenson R and Berenson J (1995) Transplantation of CD34+ peripheral blood progenitor cells after high-dose chemotherapy for patients with advanced multiple myeloma. *Blood* **96**: 390–397

Schmitz N. Linch DC. Dreger P. Goldstone AH. Boogaerts MA. Ferrant A. Demuynck HMS. Link H. Zander A. Barge A and Borkett K (1996) Randomised trial of filgrastim-mobilised peripheral blood progenitor cell transplantation versus autologous bone-marrow transplantation in lymphoma patients. *Lancet* **347**: 353–357

Shpall E. Jones R. Bearman SI. Franklin WA. Archer PG. Curiel T. Bitter M. Clarr HN. Stemmer SM. Purdy M. Meyers SE. Hami L. Taffs S. Heimfeld S. Hallagan J and Berenson RJ (1994) Transplantation of enriched CD34-positive autologous marrow into breast cancer patients following high-dose chemotherapy: influence of CD34-positive peripheral blood progenitors and growth factors on engraftment. *J Clin Oncol* **12**: 28–36

Shpall EJ and Jones RB (1994). Release of tumor cells from bone marrow. *Blood* **83**: 623–625

Shpall EJ. Bast Jr RC. Joines WT. Jones RB. Anderson I. Johnston C. Eggleston S. Tepperberg M. Edwards S and Peters WP (1991*a*). Immunomagnetic purging of breast cancer from bone marrow for autologous transplantation. *Bone Marrow Transpl* **7**: 145–151

Shpall EJ. Jones RB. Bast RC. Rosner GL. Vandermark R. Ross M. Affonti M. Johnston C. Eggleston S. Tepperburg M. Coniglio D and Peters WP (1991*b*) 4-Hydroperoxycyclophosphamide purging of breast cancer from bone marrow for autologous transplantation. *J Clin Oncol* **9**: 85–93

Shpall E. LeMaistre CF. Holland K. Ball E. Jones RB. Saral R. Jacobs C. Heimfeld S. Berenson R and Champlin R (1997) A prospective randomized trial of buffy-coat versus CD34-selected autologous bone marrow support in high-risk breast cancer patients receiving high dose chemotherapy. *Blood* **90**: 4313–4320

Simmons DL. Satterwaithe AB. Tenen DG and Seed B (1992) Molecular cloning of a cDNA encoding CD34, a sialomucin of human hematopoietic stem cells. *J Immunol* **92**: 267–271

Somlo G. Sniecinski I. Odom-Maryom T. Nowicki B. Chow W. Hamasaki V. Leong L. Margolin K. Morgan RJ. Raschko J. Shibata S. Tetef M. Molina A. Berenson RJ. Forman SJ and Doroshow JH (1997) Effect of CD34+ selection and various schedules of stem cell reinfusion and granulocyte colony-stimulating factor priming on hematopoietic recovery after high-dose chemotherapy for breast cancer. *Blood* **89**: 1521–1528

Tannock IF. Boyd NF. DeBoer G. Erlichman C. Fine S. Larocque G. Mayers C. Perrault D and Sutherland H (1988) A randomized trial of two levels of cyclophosphamide, methotrexate, and fluorouracil chemotherapy for patients with metastatic breast cancer. *J Clin Oncol* **6**: 1377–1387

To LB. Haylock DN. Simmons PJ and Juttner CA (1997) The biology and clinical uses of blood stem cells. *Blood* **89**: 2233–2258

van de Wall E. Richel DJ. Holtkamp MJ. Slaper-Cortenbach ICM. van der Schoot. CE. Dalesio O. Nooijen WJ. Schornagel JH and Rodenhuis S (1994) Bone marrow reconstitution after high-dose chemotherapy and autologous peripheral blood progenitor cell transplantation: effect of graft size. *Ann Oncol* **5**: 795–802

Wallerstein R. Spitzer G. Dunphy F. Huan S. Hortobagyi G. Yau J. Buzdar A. Holmes R. Theriault R. Ewer M. leMaistre CF. Dicke K and Deisseroth A (1990) A phase II study of mitoxantrone, etoposide and thiotepa with autologous marrow support for patients with relapsed breast cancer. *J Clin Oncol* **8**: 1782–1788

Weaver C. Hazelton B. Birch R. Palmer P. Allen C. Schwartzberg L and West W (1995) An analysis of engraftment kinetics as a function of the CD34 content of peripheral blood progenitor cell collections in 692 patients after the administration of myeloablative chemotherapy. *Blood* **86**: 3961–3969

Williams SF. Lee WJ. Bender JG. Zimmerman T. Swinney P. Blake M. Carreon J. Schilling M. Smith S. Williams DE. Oldham F and Van Epps D (1996) Selection and expansion of peripheral blood CD34+ cells in autologous stem cell transplantation for breast cancer. *Blood* **87**: 1687–1691

Wood WC. Budman DR. Korzun AH. Cooper MH. Younger J. Hart RD. Moore A. Ellerton JA. Norton L. Ferree CR. Ballow AC. Frei EI and Henderson IC (1994) Dose and dose intensity of adjuvant chemotherapy for stage II, node positive early breast cancer. *N Engl J Med* **330**: 1260–1266